# Ontogeny of Neuro-Insular Complexes and Islets Innervation in the Human Pancreas

**DOI:** 10.3389/fendo.2014.00057

**Published:** 2014-04-22

**Authors:** Alexandra E. Proshchina, Yulia S. Krivova, Valeriy M. Barabanov, Sergey V. Saveliev

**Affiliations:** ^1^Laboratory of Nervous System Development, Scientific Research Institute of Human Morphology, Department of Medical Biological Sciences, Russian Academy of Medical Science, Moscow, Russia

**Keywords:** pancreas, innervation, neuro-insular complexes, NSE, S100 protein, islets, morphogenesis, fetal

## Abstract

The ontogeny of the neuro-insular complexes (NIC) and the islets innervation in human pancreas has not been studied in detail. Our aim was to describe the developmental dynamics and distribution of the nervous system structures in the endocrine part of human pancreas. We used double-staining with antibodies specific to pan-neural markers [neuron-specific enolase (NSE) and S100 protein] and to hormones of pancreatic endocrine cells. NSE and S100-positive nerves and ganglia were identified in the human fetal pancreas from gestation week (gw) 10 onward. Later the density of S100 and NSE-positive fibers increased. In adults, this network was sparse. The islets innervation started to form from gw 14. NSE-containing endocrine cells were identified from gw 12 onward. Additionally, S100-positive cells were detected both in the periphery and within some of the islets starting at gw 14. The analysis of islets innervation has shown that the fetal pancreas contained NIC and the number of these complexes was reduced in adults. The highest density of NIC is detected during middle and late fetal periods, when the mosaic islets, typical for adults, form. The close integration between the developing pancreatic islets and the nervous system structures may play an important role not only in the hormone secretion, but also in the islets morphogenesis.

## Introduction

The pancreas is well innervated with the autonomic nervous system in various species. Cajal described a rich innervation of blood vessels and of the acinar part of the pancreas. The works of Gentes and Pensa demonstrated a network of nerve fibers in the endocrine part of the pancreas in some mammals (rat, cat, and dog) [for references see Ref. (1–3)].

The autonomic nervous system regulates the secretion of hormones from the islets of Langerhans, thus impacting glucose metabolism. The innervation of the pancreatic islets by postganglionic adrenergic and cholinergic axon terminals has been extensively studied [for references see Ref. (4, 5)]. Sympathetic neural cell bodies are located in the superior mesenteric and celiac ganglia and are components of the splanchnic nerve. Parasympathetic innervation is derived from the vagus nerve (6, 7). The pancreas is richly innervated by preganglionic vagal fibers. Autonomic nerves synapse on to intrapancreatic ganglia clusters of neurons that are spread in a connective plexus throughout the pancreas in mice, rats, cats, rabbits and guinea pigs [for references see Ref. (5)].

However, the precise innervation patterns of the islets are unknown, particularly in human. The literature data indicates that few nerve fibers are found in the pancreatic islets in adult humans in comparison with rodents (3, 8–10). However, the human pancreas receives an extensive innervation showing peculiar growth dynamics during the gestation. Nervous structures are associated with the endocrine and exocrine human pancreas similar to findings in other mammalian species during the fetal period (11). In our previous study, the rich innervation of the human fetal islets was reported (12).

In pancreata of many mammals, including rodents (mice, rats), there are complexes formed by endocrine cells of the pancreatic islets and neural structures (nerve fibers, nerve cell bodies), the so-called neuro-insular complexes (NIC) (2, 3, 13–16). Simard (17) investigated the pancreata of men of different ages and found such complexes in all specimens examined [for references see Ref. (18)]. Two types of NIC were discerned by Fujita (18): type I (NIC I), which represents the gathering of islet cells and ganglionic cells; and type II (NIC II), characterized by the intimacy of islet cells and bundles of nerve fibers. Later, the series of neuroendocrine structures was recognized and their classification was proposed (4) (Table 1).

**Table 1:** **The classification of pancreatic neuroendocrine structures (4)**.

Autonomic ganglion	No endocrine cells
Neuro-insular complex type I	Few endocrine cells in ganglion
	Few ganglionic cells in islet
Neuro-insular complex type II	Single or a few endocrine cells associated with a bundle of nerve fibers
	Heavily innervated islet tissue
Classical islet of Langerhans	Innervated
	Not innervated

In addition, glial (Schwann) cells were detected on the periphery of the pancreatic islets in several mammals (mice, rats, rabbits, etc.) using electron microscopy and immunohistochemistry. These cells were immunoreactive to the S100 protein and glial fibrillary acidic protein (GFAP) (2, 19).

These apparent contradictory observations between mammalian, fetal, and adult human islets innervation led us to study the islets innervation using a set of immunohistochemical markers such as the antibodies to hormones of pancreatic endocrine cells and to pan-neural markers. The lack of information about the structure and development of NIC makes this study compelling. To our knowledge, this is the first quantitative analysis of the NIC number in the human pancreas at different developmental stages.

## Materials and Methods

The present study was performed on autopsied pancreatic specimens from 33 human fetuses and infants (10–40 weeks of gestation) and 15 adults (aged 30–91 years old) from the collection of the Laboratory of Nervous System Development at the Research Institute of Human Morphology Russian Academy of Medical Science, Moscow. Gestational age was determined on the basis of time since the last menstrual period and the measured Crown–Rump Length and biparietal diameter by ultrasonography. Additionally, the determination of fetal age was performed in accordance with morphometric tables in the first trimester of pregnancy (20). The study was approved by a local Ethics Committee of Institute of Human Morphology, Moscow. Tissue specimen collection and handling were performed in accordance with Russian laws.

The material was fixed in 10% acidic formalin, neutral formalin (4% paraformaldehyde in 0.1 M phosphate buffer, pH 7.5), or Bouin’s fluid. The pancreata with the part of gastrointestinal tract [for the fetuses of 10 up to 23 gestation week (gw)] or pieces of the pancreatic body and tail (the human fetuses after gw 24, adults) were dehydrated, embedded in paraffin and sectioned.

The streptavidin–biotin immunohistochemical technique was performed on samples. Ten-micrometers-thick sections were dewaxed, rehydrated, and treated with 3% solution of H_2_O_2_ to block endogenous peroxidase. The specific antibodies for pancreatic hormones and the pan-neuronal markers are shown in Table 2. Reactions were detected with UltraVision Detection System anti-polyvalent with diaminobenzidine as a chromogen (Thermo Fisher Scientific Inc., Fremont, CA, USA).

**Table 2:** **Panel of antibodies used**.

Antibody specificity	Origin	Dilution	Incubation	Source
Insulin	Mouse	1:400	30 min, RT	Thermo Fisher Scientific Inc., Fremont, CA, USA
Insulin	Rabbit	1: 400	30 min, RT	Santa Cruz Biotechnology, Inc., Heidelberg, Germany
Glucagon	Rabbit	1:100	30 min, RT	Thermo Fisher Scientific Inc., Fremont, CA, USA
NSE	Mouse	1:200	30 min, RT	Thermo Fisher Scientific Inc., Fremont, CA, USA
S100 protein	Rabbit	1:100	30 min, RT	Thermo Fisher Scientific Inc., Fremont, CA, USA

*NSE, neurone-specific enolase; RT, room temperature*.

The double immunostaining was performed on serial sections in all five possible combinations. The MultiVision Polymer Detection System: MultiVision anti-rabbit/HRP + anti-mouse/AP polymers (Thermo Fisher Scientific Inc., Fremont, CA, USA) was used to identify the antigens.

Negative control sections, in which the primary antibody was omitted, were used for each case in every immunostaining run. The reactions on the human fetal pancreata in the late stages of development (30–40 gw) or on the samples of the adults’ pancreas were used as positive controls.

The count of NIC number was performed to demonstrate the dynamics of their distribution during various stages of human development. All of non-overlapping observation fields were selected within sections of the fetal pancreata stained with antibody against S100 and insulin. Seven random non-overlapping observation fields were selected in adult pancreatic sections. Each field was captured with a Sony digital camera (SSc–Dc50P) mounted on a Leica DM LS light microscope using a 10× objective and saved. The NIC of both types were counted on each photomicrographs.

The classification of the fetal period in which it is divided in four stages (prefetal, early fetal, middle fetal, and late fetal periods) was used (21). Thus, we divided all material into five groups for the statistical analysis (Table 3). A software statistical package was used (Statistica 6.0, Statsoft Inc., Tulsa, OK, USA). Data were expressed as means ± standard error of the mean (SEM). A non-parametric tests (Kruskal–Wallis and Mann–Whitney tests) were used, because values did not follow a Gaussian distribution. The *P*-value was considered significant if <0.05.

**Table 3:** **Number of neuro-insular complexes during various stages of human development (mean ± SEM)**.

	*N* of fetuses	Fields of observation	NIC I	NIC II
Prefetal period (gw 10–12)	7	7	0.0000 ± 0.0000	0.0000 ± 0000
Early fetal period (gw 13–20)	16	55	0.3272 ± 0.0901	1.0909 ± 0.1765
Middle fetal period (gw 21–28)	6	41	0.4878 ± 0.0995	1.3170 ± 0.2049
Late fetal period (gw 29–40)	4	24	0.2609 ± 0.0936	3.6087 ± 0.4434
Adults	15	105	0.0694 ± 0.0302	0.3889 ± 0.0701

## Results

**Figure 1:**
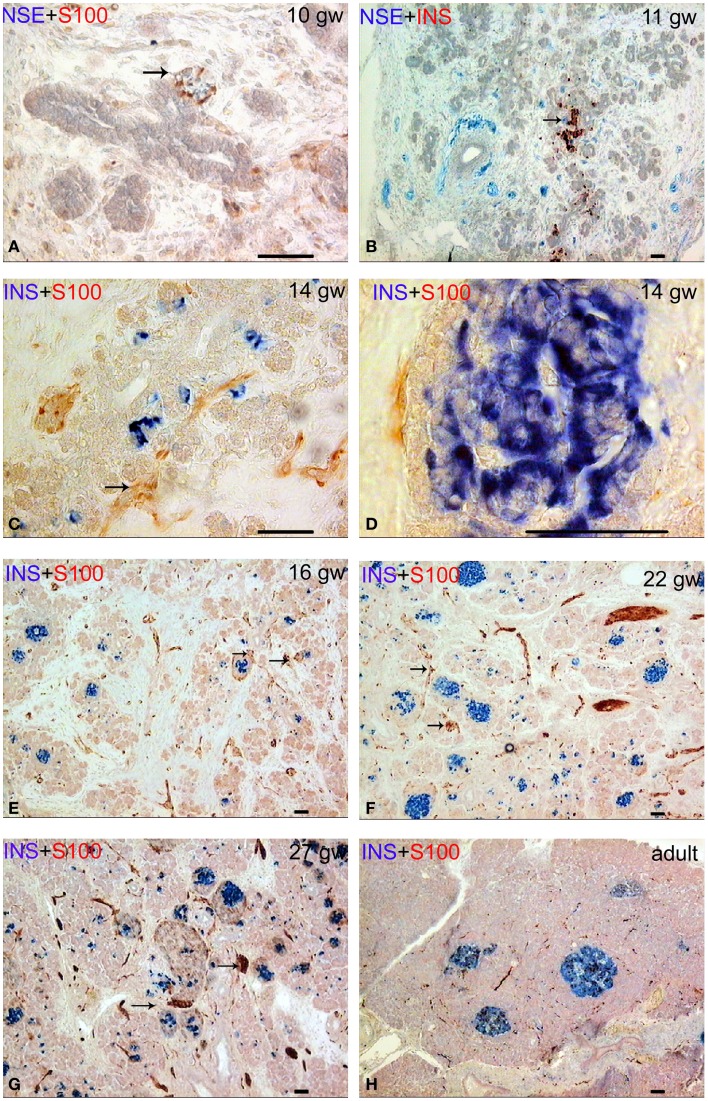
**Patterns of innervation in human fetal and adult pancreas**. Arrows indicate some ganglia. Bar = 50 mcm. **(A,B)** Innervation of the pancreas in the prefetal period showing autonomic ganglia in close proximity to the ducts. **(A)** NSE (blue) and S-100 (red) double staining. **(B)** NSE (blue) and insulin (red) double staining. **(C)** Innervation of the pancreas in early fetal period (gw 14) showing large bundles of nerve fibers and ganglia. Note that some nerve fibers are passing between two nerve ganglia. Insulin (blue) and S-100 (red) double staining. **(D)** The close proximity of the fine nerve fiber and a Langerhans’ islet at gw 14. Insulin (blue) and S-100 (red) double staining. **(E–G)** The consequent increase of pancreatic innervation in early and middle fetal periods. Insulin (blue) and S-100 (red) double staining. **(H)** Innervation of the adult pancreas. Insulin (blue) and S-100 (red) double staining.

In human fetuses from the gw 10 up to the gw 13, the pancreas consisted of a system of coupled ducts, composed of simple tubular epithelial cells. The immunopositive reaction for insulin and glucagon was found in isolated epithelial cells of the ducts in the central area of the pancreatic body in all of the investigated 10-week fetuses. At gw 10, the S100- and neuron-specific enolase (NSE)-positive nerve ganglia and large bundles of nerve fibers were located in the pancreatic mesenchyme between pancreatic ducts. Ganglia were often in close proximity to the ducts (Figures 1A,B). The neurons in the ganglia were predominantly NSE-positive and the glial cells were S100-positive. NSE-containing endocrine cells were revealed from gw 12 onward.

The lobules of the pancreas started to form during gw 14. The islets of Langerhans and innervation of the endocrine part of human pancreas also started to form at this time, in early fetal period (Figures 1C,D). The nerve fibers passing between two nerve ganglia were found in gw 14 in fetuses (Figure 1C). Few fine S100-positive fibers were observed on the periphery of some islets (Figure 1D) starting at gw 14.

Innervation of the pancreas in the 16 gw fetuses was more branched in comparison with the gw 10–14 (Figure 1E). Large nerve bundles were located in the interlobular connective tissue. Nerve fibers and ganglia were detected within the lobules. Nerve fibers entered the pancreatic lobules in association with the blood vessels or separately. Ganglionic neurons connected with the nerve fibers or directly with the islets (Figure 1E). In middle and late fetal periods, the density of the network of S100 and NSE-positive fibers increased (Figures 1F,G).

In adults, this network was sparse (Figure 1H). It should be noted that the total number of S100+ and NSE+ nerve fibers was comparable in the samples of the fetal pancreas. In adults, the NSE-positive reaction was observed in most of the endocrine cells and in large bundles of nerve fibers. Fine network of the nervous fibers and only a few cells in the endocrine pancreas were identified in adult pancreata with the antibody to S100 protein.

**Figure 2:**
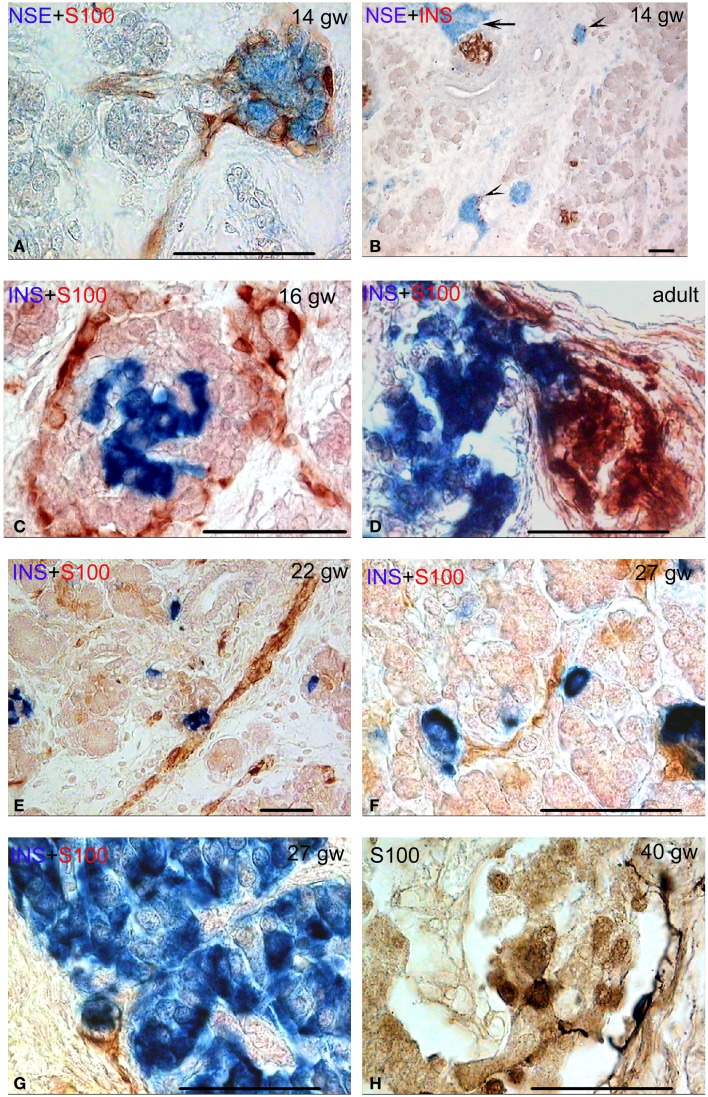
**Different types of connectivity between nervous system structures and pancreatic fetal and adult islets**. Bar = 50 mcm. **(A)** Autonomic ganglion (no endocrine cells) in human pancreas. NSE (blue) and S-100 (red) double staining. **(B)** Neuro-insular complexes type Ia (few endocrine cells in ganglion, arrow heads) and neuro-insular complex type I (the connection of the ganglion and the islet, arrow). NSE (blue) and insulin (red) double staining. **(C,D)** Neuro-insular complexes type I at various stages of human development (the connection of the ganglion and the islet). Insulin (blue) and S-100 (red) double staining. **(E–G)** Neuro-insular complex type IIa (single or a few endocrine cells associated with a bundle of nerve fibers). Insulin (blue) and S-100 (red) double staining. **(H)** S100-positive cells on the margin and within the islet. Note two cells with processes on the islet margin. S-100 staining.

The analysis of the islets innervation has shown that the pancreas contained different types of interconnection between structures of the nervous system and endocrine cells from gw 14 onward. As mentioned above, numerous nerve ganglia were detected in the sections of the developing human pancreas (Figure 2A). They were located in the interlobular connective tissue from gw 14. In the intralobular connective tissue, they were detected from gw 16 onward.

Frequently, such ganglia were integrated with a small number of insulin- or glucagon-containing cells, forming NIC type IA (Figure 2B). The connection of the ganglion directly with the islets was identified more often compared to NIC IA. Ganglionic neurons were located in the interlobular or intralobular connective tissue. Islets were also located in the same places or at the periphery of lobules (Figures 1E–G and 2C). NIC type IB were not revealed.

The number of NIC1 was higher in the early and middle fetal periods than it was in the prefetal period (Table 3) and decreased in the late fetal period. In the adults pancreata, NIC type 1 were sparse (Figure 2D). When present, they were located in the connective tissue. The number of NIC I differed significantly between adults and fetal periods (*P* < 0.01 in Kruskal–Wallis test and Table 4A).

**Table 4:** ***P*-values in the Mann–Whitney tests of the variance of NIC numbers during various stages of human development**.

**A: NIC I**				
Prefetal period				
0.1537	Early fetal period			
**0.0386**	0.919	Middle fetal period		
0.374	0.9591	0.2187	Late fetal period	
0.474	**0.0062**	**0.0000**	**0.013**	Adults
**B: NIC II**				
Prefetal period				
**0.0035**	Early fetal period			
**0.0018**	0.3248	Middle fetal period		
**0.0001**	**0.0000**	**0.0000**	Late fetal period	
0.0715	**0.0002**	**0.0000**	**0.0000**	Adults

*A value was considered significant if the *P*-value was <0.05. Significant values are presented in bold*.

NIC type II were also revealed. Endocrine cells and islets were integrated into the nerve bundle (Figures 1C and 2E), or were innervated by individual nerve fibers (Figures 2F,G). We have also observed complexes consisting of a few islets connected to each other by nerve fibers or integrated in a single ganglion. A maximum number of NIC II was obtained in the late fetal period (Table 3). In the adult pancreata, the number of NIC II was significantly reduced compared to early, middle, and late fetal periods (Table 4B).

Additionally, S100-positive cells were identified both in the periphery and within some of the islets (Figures 1E–G and 2C–E) starting from gw 14. Such cells were detected also in the pancreatic islets of newborn (Figure 2H) and adults. The cells, showing immuno-positivity for the protein S100, had different shapes (round, flat, or multipolar cells with processes). Cells with processes were located mostly in the periphery of islets.

## Discussion

Recently, pancreatic innervation is being viewed with increasing interest with respect to pancreatic disease. According to some authors, the structures of the nervous system (nerves, neurons, and peri-insular glia) in the pancreas are the primary target of the autoimmune attack in diabetes type one (19, 22). A decrease in islet innervation has been shown to be an early target in diabetes (23, 24). At the same time, relatively little is currently known about the dynamics of pancreatic innervation during development and disease (11).

Innervation of the human islets differs from that of the murine islets. According to the literature data, human islets are innervated less then islets of other investigated mammals (3, 8–10). For example, in mouse islets, parasympathetic and sympathetic axons innervate beta, alpha, and delta cells, but in human islets endocrine cells are barely innervated. Few nerve fibers are found in the adults’ pancreatic islets (10).

In general, our data about the developmental dynamics of human pancreatic innervation are in agreement with previously reported data (11, 12). Our results show that innervation of human islets forms from gw 14 onward. It differs from data obtained in mammals (rodents). According to literature data, innervation of the pancreatic islets in rodents (mouse, mongolian gerbil, golden hamster) was observed in the first week after birth (15, 25, 26). The contrasting results may be due to differences in morphogenesis of the islets in rodents compared to humans. In rodents, the formation of pancreatic islets was observed in the first 2 weeks after birth (15, 27–29). The insulin-containing cells of rodents occupy the central position in pancreatic islets and the glucagon-containing cells are localized at the periphery. In human pancreatic islets, a mosaic distribution of islet cells has been predominantly observed (30, 31). In small human islets (40–100 μ in diameter), the insulin-containing cells are located in a core position, and the glucagon- and somatostatin-containing cells are located in a mantle, like in rodents. In islets with a diameter over 100 μ the somatostatin- and glucagon-containing cells have a similar mantle position but they are found also along the vessels that penetrate and branch inside the islets (32, 33). Although rodent islet development is well studied, little is known about formation of the mature islet in humans. (34, 35).

Modern ideas about the development of the endocrine pancreas are mainly limited by the data of the first differentiation of endocrine cells and formation of the murine type of islets in the early stages of development (weeks 7–14). Later gestational periods have been investigated only in a few studies. In a recent study (12), we have shown that the human islet formation is gradual during gestation. Typical for adults, the large islets are identified from gw 25–27 onward. Their number increases to birth. It is important to note that there are various forms of organization of the fetal islets at the same time. As stated above, the fetal pancreas is more innervated than in adults. We propose that the appearance of new types of islet and the islet innervation are interconnected.

Additionally, the analysis of the innervation of pancreatic islets shows that there are several types of complexes between the pancreatic islets and the nervous system. The NIC I (endocrine cells associated with the bodies of neurons and their processes) and NIC II (endocrine cells associated only with nerve fibers) are characteristic for the pancreata of the most investigated mammals. In our previous study (12), we have described the NIC I and NIC II in the human fetal pancreas from the 14–15 weeks of development and during all of the following fetal periods. The classification of Böck (4) is incomplete as it does not include a variant of the neuro-insular complex type I revealed in various mammalian species, where the ganglion is associated directly with an islet. Such complexes were also described during prenatal development in human (12). In this study, we confirm and expand on the previous data, using a multi-labeling technique with the anti S100- and anti NSE-antibody. We reveal different types of NIC in human pancreas. Moreover, NIC consisting of several interconnecting islets connected to nerve fibers or associated with the ganglion were detected. Such complexes in the human pancreas have not been described previously, but are characteristic of some of the mammalian species (16). The neuroendocrine complexes are numerous in the pancreas in middle and late fetal periods during the active morphogenesis of the endocrine part, but are less common in the adult pancreas.

Additionally, the S100 protein-containing cells were detected in the human islets of Langerhans. The same cells were previously described by Lászik et al. (36). In the murine pancreas, Sunami et al. (2) have identified the S100-positive cells on the margin of the islets as if delimiting the islet and the exocrine parenchyma. According to these authors, the distribution and morphological features of S100 immunoreactive cells are closely similar to those of the interstitial cells of Cajal. Their findings indicate that these cells are the peculiar-shaped Schwann cells. In our study, the S100-positive cells differ in shape and localization. We propose that the S100-positive cells located on the periphery of islets are a part of NIC. However, there is no clarity of their nature.

According to Fujita (18), the series of neuroendocrine structure in the pancreas are: autonomic ganglion without islet cells, NIC I and islet without ganglion cells, indicating gradual transformation from one to the other. Furthermore, an islet devoid of neuron somata may contain bundles of axons, thus forming NIC II (37). However, the endodermal origin of pancreatic endocrine cells is now understood. For that very reason, the close intimacy between endocrine cells and neuronal tissue remains a remarkable fact [for references see Ref. (4)]. It is known that the endocrine cells of pancreatic islets are similar to nervous cells in a number of biochemical and physiological characteristics. Pancreatic islet cells share similarities with neurons in expressing neural markers, including GFAP, glutamic acid decarboxylase (GAD), tyrosine hydroxylase (TH), neuropeptide Y (NPY), NSE, neural cell adhesion molecules (NCAM), the beta-III tubulin, the synaptophysin, the nerve growth factors and their receptors, chromogranin A, and others (12, 19, 38–43). Alpha-cells contain acetylcholine (44). In addition, a number of transcription factors, which are characteristic of the nervous system such as Ngn3 (neurogenin3), Beta2/NeuroD, etc., are expressed during the differentiation of pancreatic endocrine cells.

We assume that the expression of a part of the neuroendocrine markers in islets cells is under the control of the nervous system and starts after the innervation of the islets. The temporal coincidence of the start of the islets formation, neuroendocrine expression (12), and the pancreatic innervation (11) confirms this hypothesis.

The idea of a regulatory role of the nervous system in the endocrine secretion is commonly accepted now (45). A potential synchronizing mechanism is neural input from intrapancreatic ganglia.

Our results show that the density of nerves, ganglion, and NIC is higher in the fetal pancreas compared to adults. It is obvious that the observed differences between the nervous apparatus of the pancreata in fetuses and adults may have functional significance for the morphogenesis of the islets. After experimental chemical destruction of insulin-containing cells in newborn mice, regeneration of these cells was accompanied by recovery of the sympathetic innervation (15). This data confirms the hypothesis of the functional relationship of the nervous and endocrine systems in the pancreas. There is also the hypothesis of a mechanical role of the nervous system in the formation of pancreatic islets and that the autonomous nerves can permeate protrusion groups of endocrine cells to form the path for migration of endocrine cells (46).

The highest density of NIC of both types is detected in the middle and late fetal periods, when the mosaic islets, typical for adults, form. The close integration between the developing pancreatic islets and nervous system structures suggest that the neuroendocrine interactions may affect not only the hormone secretion but also the islet morphogenesis. Knowing the roles of NIC in pancreatic islets architecture, development and functional maturation may lead us to new perspectives for understanding and treatment of diabetes mellitus. Further investigations will be necessary to assess the specific role of this tight neuroendocrine association.

## Author Contributions

Conceived and designed the experiments: Alexandra E. Proshchina, Yulia S. Krivova, Valeriy M. Barabanov, Sergey V. Saveliev. Performed the experiments: Alexandra E. Proshchina, Yulia S. Krivova, Valeriy M. Barabanov. Analyzed the data: Alexandra E. Proshchina, Yulia S. Krivova, Valeriy M. Barabanov. Contributed reagents/materials/analysis tools: Sergey V. Saveliev. Wrote the paper: Alexandra E. Proshchina. All of the authors of this paper have read and approved the version submitted.

## Conflict of Interest Statement

The authors declare that the research was conducted in the absence of any commercial or financial relationships that could be construed as a potential conflict of interest.

## References

[1] Coupland RE The innervation of pancreas of the rat, cat and rabbit as revealed by the cholinesterase technique. J Anat (1958) 92:143–9 13513506 PMC1244972

[2] Sunami E Kanazawa H Hashizume H Takeda M Hatakeyama K Ushiki T Morphological characteristics of Schwann cells in the islets of Langerhans of the murine pancreas. Arch Histol Cytol (2001) 64:191–201 10.1679/aohc.64.191 11436989

[3] Pour PM Saruc M The pattern of neural elements in the islets of normal and diseased pancreas and in isolated islets. JOP (2011) 12:395–403 21737903

[4] Böck P Fine structure of the neuro-insular complex type II in the cat. Arch Histol Jpn (1986) 49:189–97 10.1679/aohc.49.189 3532999

[5] Cerf ME Islet organogenesis, angiogenesis and innervation. Cell Biol Int (2011) 35:1065–78 10.1042/CBI20100780/21999312

[6] Salvioli B Bovara M Barbara G De Ponti F Stanghellini V Tonini M Neurology and neuropathology of the pancreatic innervation. JOP (2002) 3:26–33 11884764

[7] Cabrera-Vásquez S Navarro-Tableros V Sánchez-Soto C Gutiérrez-Ospina G Hiriart M Remodelling sympathetic innervation in rat pancreatic islets ontogeny. BMC Dev Biol (2009) 17(9):200934 10.1186/1471-213X-9-34 19534767 PMC2711085

[8] Fink T Di Sebastiano P Bochlerj M Beger HG Weihe E Growth-associated protein-43 and protein gene-product 9,5 innervation in human pancreas: changes in chronic pancreatitis. Neuroscience (1994) 63:249–66 10.1016/0306-4522(94)90020-5 7898650

[9] Castorina S Romeo R Marcello MF Immunohistochemical study of intrinsic innervation in the human pancreas. Boll Soc Ital Biol Sper (1996) 72:1–7 8868108

[10] Rodriguez-Diaz R Abdulreda MH Formoso AL Gans I Ricordi C Berggren P-O Innervation patterns of autonomic axons in the human endocrine pancreas. Cell Metab (2011) 14:45–54 10.1016/j.cmet.2011.05.008 21723503 PMC3135265

[11] Amella C Cappello F Kahl P Fritsch H Lozanoff S Sergi C Spatial and temporal dynamics of innervation during the development of fetal human pancreas. Neuroscience (2008) 154:1477–87 10.1016/j.neuroscience.2008.04.050 18538483

[12] Krivova YS Proshchina AE Barabanov VM Saveliev SV Development of the islets of Langerhans in the human fetal pancreas. In: Satou A Nakamura H, editors. Pancreas: Anatomy, Diseases and Health Implications. New York: Nova Biomedical (2012). p. 55–88

[13] Donev S Ultrastructural evidence for presence of a glial sheath investing the islets of Langerhans in the pancreas of mammals. Cell Tissue Res (1984) 237:343–8 10.1007/BF00217154 6383622

[14] Persson-Sjögren S Zashihin A Forsgren S Nerve cells associated with the endocrine pancreas in young mice: an ultrastructural analysis of the neuroinsular complex type I. Histochem J (2001) 33:373–8 10.1023/A:1012439510709 11758814

[15] Burris R Hebrok M Pancreatic innervation in mouse development and β-cell regeneration. Neuroscience (2007) 150:592–602 10.1016/j.neuroscience.2007.09.079 18006238 PMC2245866

[16] Krivova YS Barabanov VM Savel’eva ES Savel’ev SV Immunohistochemical detection of SNAP-25, NCAM, and insulin in the pancreas of nutria (*Myocastor coypus*). Bull Exp Biol Med (2007) 144:737–40 10.1007/s10517-007-0420-4 18683511

[17] Simard LC Les complecses neuro-insulaires. Arch Anat Micr (1937) 33:49–64

[18] Fujita T Histological studies on the neuro-insular complex in the pancreas of some mammals. Z Zselloforsch (1959) 50:94–109 10.1007/BF003426562667398

[19] Winer S Tsui H Lau A Song A Li X Cheung RK Autoimmune islet destruction in spontaneous type 1 diabetes is not β-cell exclusive. Nat Med (2003) 9:198–205 10.1038/nm818 12539039

[20] Patten BM Human Embryology. 2nd ed New York: McGraw-Hill Book Co., Inc (1953).

[21] Milvanov AP Saveliev SV Rational periodization of prenatal human development and methodical aspects of embryology. In: Milovanov AP Saveliev SV, editors. Prenatal Human Development. Moscow: MDV (2006). p. 21–32

[22] Carrillo J Puertas MC Alba A Ampudia RM Pastor X Planas R Islet-infiltrating B-cells in nonobese diabetic mice predominantly target nervous system elements. Diabetes (2005) 54:69–77 10.2337/diabetes.54.1.69 15616012

[23] Saravia F Homo-Delarche F Is innervation an early target in autoimmune diabetes? Trends Immunol (2003) 24:574–9 10.1016/j.it.2003.09.010 14596878

[24] Persson-Sjogren S Holmberg D Forsgren S Remodeling of the innervation of pancreatic islets accompanies insulitis preceding onset of diabetes in the NOD mouse. J Neuroimmunol (2005) 158:128–37 10.1016/j.jneuroim.2004.08.019 15589046

[25] Cegrell L The postnatal occurrence of biogenic monoamines in pancreatic islets of golden hamsters. Acta Endocrinol (Copenh) (1975) 78:289–93 1090102 10.1530/acta.0.0780289

[26] Thomas NW Findlay JA Innervation of the endocrine pancreas in the mongolian gerbil (*Meriones unguiculatus*). In: Coupland RE Forssmann WG, editors. Peripheral Neuroendocrine Interactions. New York: Springer-Verlag (1978). p. 134–43

[27] Cirulli V Beatns D Rutishauser U Halban PA Orci L Rouiller DG Expression of neural cell adhesion molecule (N-CAM) in rat islets and its role in islet cell type segregation. J Cell Sci (1994) 107:1429–36 7962186 10.1242/jcs.107.6.1429

[28] Esni F Taljedal IB Perl AK Cremer H Christofori G Semb H Neural cell adhesion molecule (N-CAM) is required for islet cell type segregation and normal ultrastructure in pancreatic islets. J Cell Biol (1999) 144:325–37 10.1083/jcb.144.2.325 9922458 PMC2132899

[29] Johansson M Andersson A Carlsson P-O Jansson L Perinatal development of the pancreatic islet microvasculature in rats. J Anat (2006) 208:191–6 10.1111/j.1469-7580.2006.00520.x 16441563 PMC2100194

[30] Brissova M Flower MJ Nicholson WE Chu A Hirshberg B Harlan DM Assessment of human pancreatic islet architecture and composition by laser scanning confocal microscopy. J Histochem Cytochem (2005) 53:1087–97 10.1369/jhc.5C6684.2005 15923354

[31] Cabrera O Berman DM Kenyon NS Ricordi C Berggren P-O Caicedo A The unique cytoarchitecture of human pancreatic islets has implications for islet cell function. Proc Natl Acad Sci U S A (2006) 103:2334–9 10.1073/pnas.0510790103 16461897 PMC1413730

[32] Bosco D Armanet M Morel PH Niclauss N Sgroi A Muller YD Unique arrangement of α- and β-cells in human islets of Langerhans. Diabetes (2010) 59:1202–10 10.2337/db09-1177 20185817 PMC2857900

[33] Proshchina AE Saveliev SV Immunohistochemical study of α- and β-cell distribution in human pancreatic Langerhans islets of various types. Bull Exp Biol Med (2013) 155:798–801 10.1007/s10517-013-2255-5 24288769

[34] Scharfmann R Xiao X Heimberg H Mallet J Ravassard P Betacells within single human islets originate from multiple progenitors. PLoS One (2008) 3(10):e3559 10.1371/journal.pone.0003559 18958289 PMC2571119

[35] Jeon J Correa-Medina M Ricordi C Edlund H Diez JA Endocrine cell clustering during human pancreas development. J Histochem Cytochem (2009) 57:811–24 10.1369/jhc.2009.953307 19365093 PMC2728126

[36] Lászik Z Krenács T Dobó E S-100 protein immunoreactivity in human islets of Langerhans. Acta Morphol Hung (1989) 37:117–24 2518343

[37] Serizawa Y Kobayashi S Fujita T Neuro-insular complex type I in the mouse. Re-evaluation of the pancreatic islet as a modified ganglion. Arch Histol Jpn (1979) 42:389–94 10.1679/aohc1950.42.389 395924

[38] Von Dorsche HH Fält K Hahn HJ Reiher H Neuron-specific enolase (NSE) as a neuroendocrine cell marker in the human fetal pancreas. Acta Histochem (1989) 85:227–8 10.1016/S0065-1281(89)80073-X 2500832

[39] Kim J Richter W Aanstoot HJ Shi Y Fu Q Rajotte R Differential expression of GAD65 and GAD67 in human, rat, and mouse pancreatic islets. Diabetes (1993) 42:1799–808 10.2337/diab.42.12.1799 8243826

[40] Teitelman G Alpert S Polak JM Martinez A Hanahan D Precursor cells of mouse endocrine pancreas co-express insulin, glucagon and the neuronal proteins tyrosine hydroxylase and neuropeptide Y, but not pancreatic polypeptide. Development (1993) 118:1031–9 7903631 10.1242/dev.118.4.1031

[41] Sandberg A Pour PM Expression of nerve growth factors in pancreatic neural tissue and pancreatic cancer. J Histochem Cytochem (2001) 49:1205–10 10.1177/002215540104901002 11561004

[42] Tezel E Nagasaka T Nomoto S Sugimoto H Nakao A Neuroendocrine-like differentiation in patients with pancreatic carcinoma. Cancer (2000) 89:2230–6 10.1002/1097-0142(20001201)89:11<2230::AID-CNCR11>3.0.CO;2-X 11147593

[43] Tsui H Winer S Jakowsky G Dosch H-M Neuronal elements in the pathogenesis of type 1 diabetes. Rev Endocr Metab Disord (2003) 4:301–10 10.1023/A:1025374531151 14501181

[44] Rodriguez-Diaz R Dando R Jacques-Silva C Fachado A Molina J Abdulreda MH Alpha cells secrete acetylcholine as a non-neuronal paracrine signal priming beta cell function in humans. Nat Med (2012) 17:888–92 10.1038/nm.2371 21685896 PMC3132226

[45] Ahrén B Autonomic regulation of islet hormone secretion – implications for health and disease. Diabetologia (2000) 43:393–410 10.1007/s001250051322 10819232

[46] Fujisawa M Notohara K Tsukayama C Mizuno R Okada S CD-56 positive cells with or without synaptophysin expression are recognized in the pancreatic duct epithelium: a study with adult and fetal tissues and specimens from chronic pancreatitis. Acta Med Okayama (2003) 57:279–84 14726964 10.18926/AMO/32811

